# Case Report: An Ulceration With a Stalactite Appearance on the Index Finger

**DOI:** 10.3389/fmed.2022.801086

**Published:** 2022-04-18

**Authors:** Liangliang Zhang, Yalan Dai, Mengting Lin, Qiuyun Xu, Tingting Lin, Ting Gong, Bo Cheng, Chao Ji, Donghua Cai

**Affiliations:** ^1^Department of Dermatology, The First Affiliated Hospital of Fujian Medical University, Fuzhou, China; ^2^Department of Dermatology, Shishi Municipal General Hospital, Quanzhou, China; ^3^Central Laboratory, The First Affiliated Hospital of Fujian Medical University, Fuzhou, China

**Keywords:** *Proteus mirabilis*, cutaneous exanthem, next generation sequencing, *Proteus* spp. bacteria, human infection

## Abstract

*Proteus mirabilis*, the most widespread species of all *Proteus* spp. bacteria, is proven to be one of the most universal pathogens in chronic wounds. In this case, a woman in her 40s consulted a physician about an asymptomatic ulceration with a stalactite appearance at the distal end of the index finger after she was exposed to a needle when vaccinating chickens. The patient did not response to ceftazidime. Physical examination revealed a well-demarcated violescent ulceration with a stalactite appearance at the distal end of the index finger. A biopsy of the lesion showed dense infiltration of multinucleated giant cells, histiocytes, and lymphocytes in the dermis. The result of metagenomics next-generation sequencing (NGS) showed 306 unique sequence reads of *P. mirabilis*, covering 33.49% of the nucleotide sequences. The pathogen was identified as *P. mirabilis*, which was resistant to ceftazidime. The patient was treated with ciprofloxacin hydrochloride and improved considerably. This case reported a distinctive cutaneous lesion of *P. mirabilis* on human infection and showed a successful use of NGS in *P. mirabilis*.

## Introduction

*Proteus mirabilis* is the most widespread species of *Proteus* spp. bacteria (1). Culture, biochemical tests, and multiplex PCR can be used for diagnosis of *P. mirabilis* (2–4). Metagenomics next-generation sequencing (NGS) has the potential to identify and quantify new or unexpected pathogens in challenging situations such as culture- and PCR-negative results (5–8).

## Case Report

**FIGURE 1:**
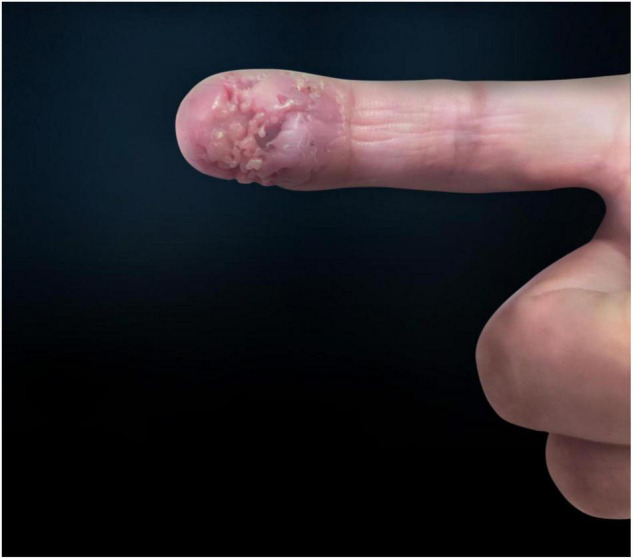
Ulceration with a stalactite appearance at the distal end of the index finger.

**FIGURE 2:**
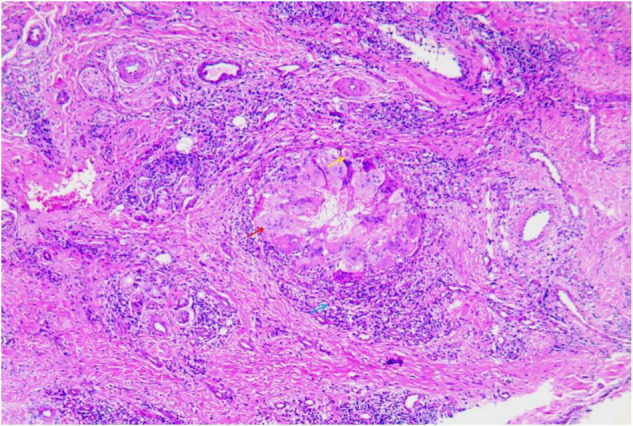
Biopsy of the lesion showed dense infiltration of multinuclear giant cells (red arrow), histiocytes (yellow arrow), and lymphocytes (blue arrow) in the dermis (hematoxylin and eosin stain, ×400).

**FIGURE 3:**
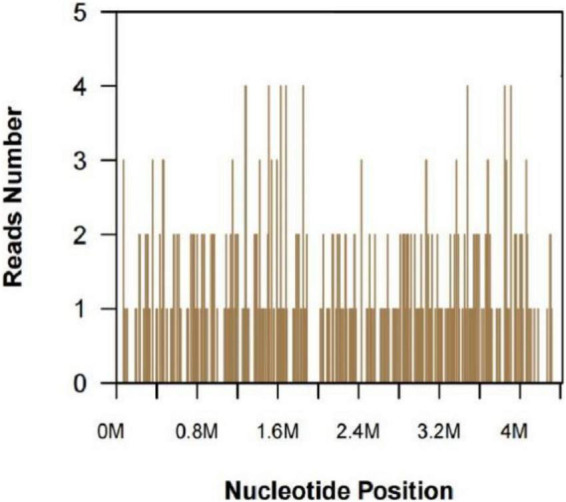
Next-generation sequencing and metagenomics analysis. *Proteus mirabilis* specific reads and its nucleotide position along the *Proteus mirabilis* genome.

A 47-year-old woman presented with an asymptomatic ulceration with a stalactite appearance at the distal of end of the index finger for 30 days. She reported no dyspnea, fever, arthralgias, or other systemic symptoms. Prior to admission to our department, the patient received ceftazidime 2 mg twice daily, but her lesion worsened. One month earlier, the patient had a needle-stick injury while vaccinating chickens. However, she did not report any exposure to the vaccine. Physical examination revealed a well-demarcated violescent ulceration with a stalactite appearance at the distal end of the index finger (Figure 1). Biopsy of the lesion showed a dense infiltration of multinucleated giant cells, histiocytes, and lymphocytes in the dermis (Figure 2). A culture performed on the cutaneous lesion was negative in this case. Metagenomics NGS of the specimen was performed (Supplementary Methods). Four negative and positive control samples were subjected to the same procedures. NGS result showed that *P. mirabilis* had 306 unique sequence reads, covering 33.49% of the nucleotide sequences (Figure 3). The pathogen was identified as *P. mirabilis*, which was resistant to ceftazidime. The patient was treated with ciprofloxacin hydrochloride (at a dose of 1.5 g three times daily for 20 days), and her lesion showed substantial improvement (Supplementary Figure 1). After 2 weeks, dermatology life quality index (DLQI) decreased rapidly from 15 to 2.

## Discussion

*Proteus mirabilis* is one of the most universal pathogens in chronic trauma, accounting for 8.0% (9). Humans were infected with *P. mirabilis* from consumption and slaughter of wild and domestic animals (mammals, birds, reptiles, amphibians, insects, and seafood) (10). The phenotypic and virulence gene characteristics in human and chicken isolates of *P. mirabilis* are similar (11). Cellulitis has been reported in broiler chickens (12). However, features of skin lesions in human *P. mirabilis* infection are rarely described. Culture is an important diagnostic tool for *P. mirabilis*, which has a characteristic swarming motility and distinct fishy smell (2). There are also both traditional and kit-based biochemical tests in addition to molecular-based multiplex PCR, which can be used for diagnosis of *P. mirabilis* (3).

In this case, the patient had a history of a needle-stick injury and a specific exanthem of asymptomatic ulceration with stalactite appearance. The antibiotic (ceftazidime) was ineffective, and the culture was negative. *P. mirabilis* may be in a viable but non-culturable (VBNC) state. It was a state of bacteria where bacteria under certain conditions of starvation or stress could enter a state of no division and, therefore, become non-culturable but in the same time keep their viability and sometimes their ability to cause infection (13). Thus, VBNC of *P. mirabilis* was a possible reason for non-culturability of the bacteria and may also explain the failure of ceftazidime treatment, which required actively dividing bacteria to performed their antimicrobial effect (14, 15).

We chose to perform mNGS to blindly detect the causative agent, as PCR required multiple tests. Besides, NGS has the potential to predict antibiotic resistance. Many resistant genes coding beta-lactamases were detected in *P. mirabilis*, including TEM genes, VEB-1 genes, OXA genes, CTX-M genes and CIT genes, with the TEM genes being the most common resistant gene (16). In conclusion, this case reported a characteristic cutaneous lesion of *P. mirabilis* after human infection and showed the successful use of NGS in *P. mirabilis*. Further studies need to enlarge the sample size to support the clinical utility of NGS metagenomic testing.

## Data Availability Statement

The datasets presented in this study can be found in the online repository at: https://www.ncbi.nlm.nih.gov/guide under accession number PRJNA813626.

## Ethics Statement

The studies involving human participants were reviewed and approved by the Medical Technology Clinical Application Ethics Committee of The First Affiliated Hospital of Fujian Medical University. The patients/participants provided their written informed consent to participate in this study. Written informed consent was obtained from the individual(s) for the publication of any potentially identifiable images or data included in this article.

## Author Contributions

LZ, YD, CJ, and DC conceptualized and designed the study. LZ and YD wrote the manuscript. TG, BC, CJ, and DC revised the manuscript critically for important intellectual content. ML, QX, and TL collected clinical pictures and analyzed the data. All authors have read and approved the final manuscript.

## Conflict of Interest

The authors declare that the research was conducted in the absence of any commercial or financial relationships that could be construed as a potential conflict of interest.

## Publisher’s Note

All claims expressed in this article are solely those of the authors and do not necessarily represent those of their affiliated organizations, or those of the publisher, the editors and the reviewers. Any product that may be evaluated in this article, or claim that may be made by its manufacturer, is not guaranteed or endorsed by the publisher.

## Supplementary Material

Supplementary Figure 1 Data of therapeutic interventions.
