# Supplementary figures and images for: Case Report: An Ulceration With a Stalactite Appearance on the Index Finger
Source: Front Med (Lausanne). 2022 Apr 18;9:801086. doi: 10.3389/fmed.2022.801086 (PMC9058116; doi:10.3389/fmed.2022.801086)

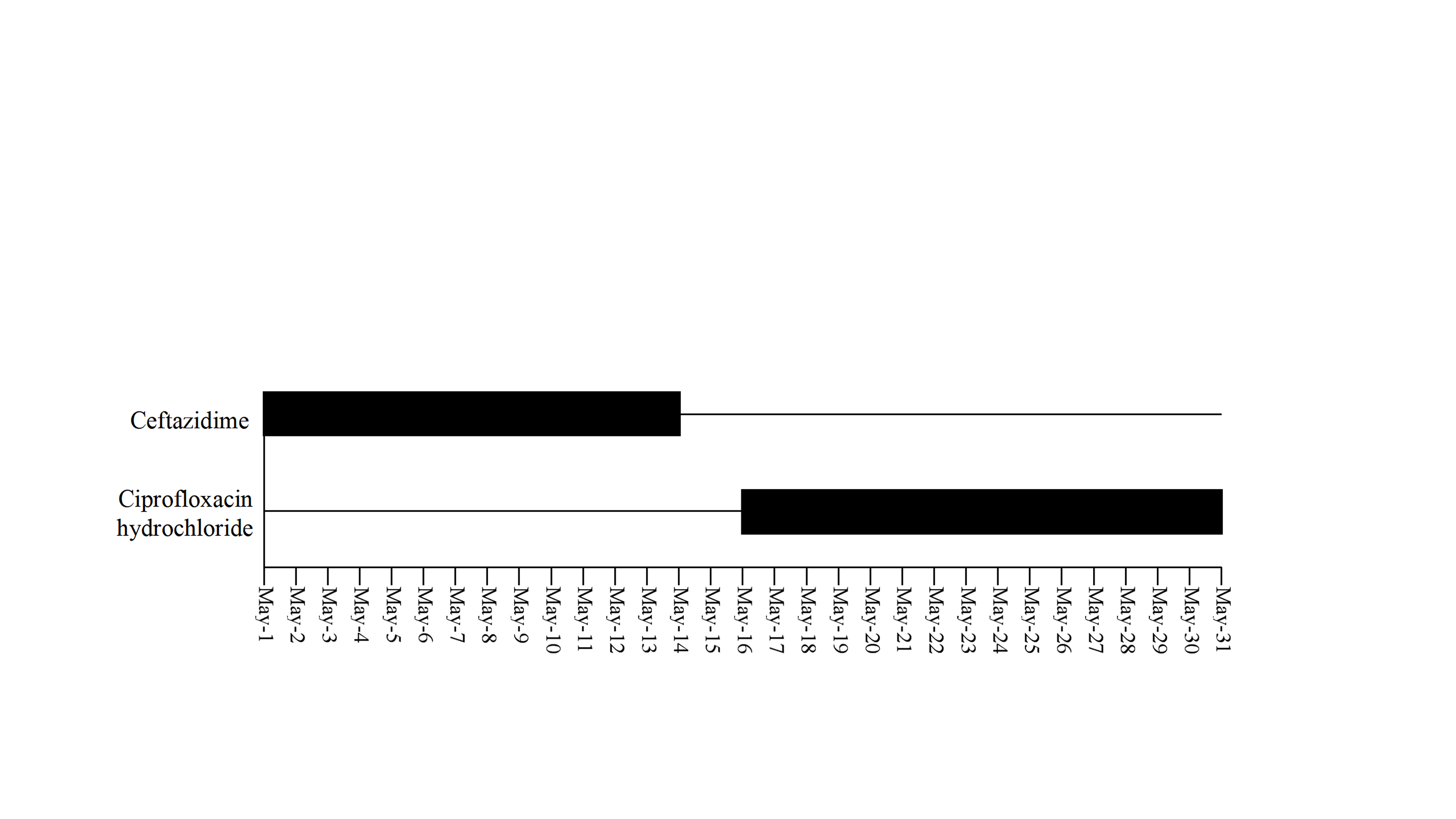

Supplement: Supplementary Figure 1 — Data of therapeutic interventions. [file Image_1.TIF]

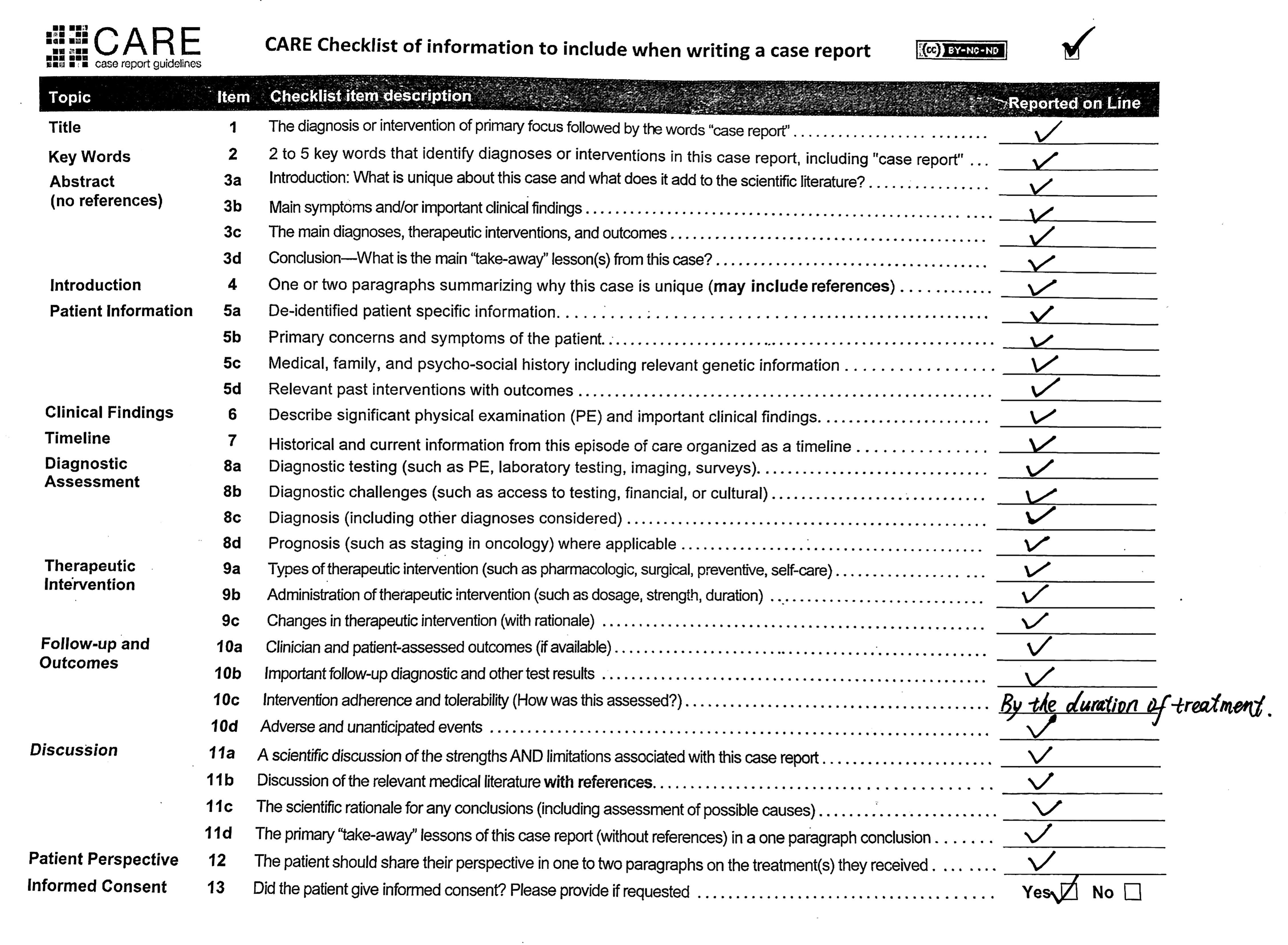

Supplement: Supplementary file 3 [file Image_2.PNG]
