# Supplementary material for: Case Report: An Ulceration With a Stalactite Appearance on the Index Finger
Source: Front Med (Lausanne). 2022 Apr 18;9:801086. doi: 10.3389/fmed.2022.801086 (PMC9058116; doi:10.3389/fmed.2022.801086)
Supplement: Supplementary file 2 [file Data_Sheet_1.pdf]

The metagenomics next generation sequencing (NGS) methods

Sample Processing and DNA Extraction

3mL cutaneous sample from patient was collected according to standard procedures. 1.5mL microcentrifuge tube with 0.6mL sample and 250μL 0.5mm glass bead were attached to a horizontal platform on a vortex mixer and agitated vigorously at 2800-3200 rpm for 30 min. Then 7.2μL lysozyme was added for wall-breaking reaction. 0.3mL sample was separated into a new 1.5mL microcentrifuge tube and DNA was extracted using the TIANamp Micro DNA Kit (DP316, TIANGEN BIOTECH) according to the manufacturer's recommendation.

Construction of DNA libraries and Sequencing

Then, DNA libraries were constructed through DNA-fragmentation, end-repair, adapter-ligation and PCR amplification. Agilent 2100 was used for quality control of the DNA libraries. Quality qualified libraries were pooled, DNA Nanoball (DNB) was made and sequenced by BGISEQ-50 /MGISEQ-2000 platform [1].

Bioinformatic analysis

High-quality sequencing data were generated by removing low-quality reads, followed by computational substraction of human host sequences mapped to the human reference genome (hg19) using Burrows-Wheeler Alignment [2]. The remaining data by removal of low-complexity reads were classified by simultaneously aligning to Pathogen metagenomics Database (PMDB), consisting of bacteria, fungi, viruses and parasites. The classification reference databases were downloaded from NCBI (ftp://ftp.ncbi.nlm.nih.gov/genomes/). RefSeq contains 4,945 whole genome sequence of viral taxa, 6,350 bacteral genomes or scaffolds, 1064 fungi related to human infection, and 234 parasites associated with human diseases.

References

[1] Y.J. Jeon, Y. Zhou, Y. Li, Q. Guo, J. Chen, S. Quan, A. Zhang, H. Zheng, X. Zhu, J. Lin, H. Xu, A. Wu, S.G. Park, B.C. Kim, H.J. Joo, H. Chen, J. Bhak, The feasibility study of non-invasive fetal trisomy 18 and 21 detection with semiconductor sequencing platform, PLoS One 9(10) (2014) e110240.

[2] H. Li, R. Durbin, Fast and accurate short read alignment with Burrows-Wheeler transform, Bioinformatics 25(14) (2009) 1754-60.
